# Correction: BRD9 Degradation Disrupts Ribosome Biogenesis in Multiple Myeloma

**DOI:** 10.1158/1078-0432.CCR-24-2112

**Published:** 2024-09-03

**Authors:** Keiji Kurata, Mehmet K. Samur, Priscilla Liow, Kenneth Wen, Leona Yamamoto, Jiye Liu, Eugenio Morelli, Annamaria Gulla, Yu-Tzu Tai, Jun Qi, Teru Hideshima, Kenneth C. Anderson

In the original version of this article (1), an incorrect FACS plot was used to represent CD138^−^ patient myeloma cells in Supplementary Fig. S1G. Supplementary Fig. S1 has been replaced with a corrected version in the latest online HTML version of the article. The authors regret this error.
